# Medication use during pregnancy, gestational age and date of delivery: agreement between maternal self-reports and health database information in a cohort

**DOI:** 10.1186/s12884-015-0745-3

**Published:** 2015-11-25

**Authors:** Federica Edith Pisa, Anica Casetta, Elena Clagnan, Elisa Michelesio, Liza Vecchi Brumatti, Fabio Barbone

**Affiliations:** Institute of Hygiene and Clinical Epidemiology, University Hospital of Udine, Via Colugna 50, 33100 Udine, Italy; Department of Medical and Biological Sciences, University of Udine, Udine, Italy; Direzione Centrale Salute, Integrazione Socio Sanitaria e Politiche Sociali, Regione Friuli Venezia Giulia Udine, Italy; INSIEL SpA, Udine, Italy; Scientific Direction, Institute for Maternal and Child Health - IRCCS “Burlo Garofolo”, Trieste, Italy; Department of Medicine, University of Trieste, Trieste, Italy

**Keywords:** Pregnancy, Medication use, Health database, Dispensing claims, Birth certificate, Agreement, Kappa, Prevalence and bias-adjusted Kappa, Pharmacoepidemiology, Questionnaires

## Abstract

**Background:**

Health databases are a promising resource for epidemiological studies on medications safety during pregnancy. The reliability of information on medications exposure and pregnancy timing is a key methodological issue. This study (a) compared maternal self-reports and database information on medication use, gestational age, date of delivery; (b) quantified the degree of agreement between sources; (c) assessed predictors of agreement.

**Methods:**

Pregnant women recruited in a prenatal clinic in Friuli Venezia Giulia (FVG) region, Italy, from 2007 to 2009, completed a questionnaire inquiring on medication use during pregnancy, gestational age and date of delivery. Redeemed prescriptions and birth certificate records were extracted from regional databases through record linkage. Percent agreement, Kappa coefficient, prevalence and bias-adjusted Kappa (PABAK) were calculated. Odds Ratio (OR), with 95 % confidence interval (95 % CI), of ≥1 agreement was calculated through unconditional logistic regression.

**Results:**

The cohort included 767 women, 39.8 % reported medication use, and 70.5 % were dispensed at least one medication. Kappa and PABAK indicated almost perfect to substantial agreement for antihypertensive medications (Kappa 0.86, PABAK 0.99), thyroid hormones (0.88, 0.98), antiepileptic medications (1.00, 1.00), antithrombotic agents (0.70, 0.96). PABAK value was greater than Kappa for medications such as insulin (Kappa 0.50, PABAK 0.99), antihistamines for systemic use (0.50, 0.99), progestogens (0.28, 0.79), and antibiotics (0.12, 0.63). Adjusted OR was 0.48 (95 % CI 0.26; 0.90) in ex- vs. never smokers, 0.64 (0.38; 1.08) in < high school vs. university, 1.55 (1.01; 2.37) in women with comorbidities, 2.25 (1.19; 4.26) in those aged 40+ vs. 30–34 years.

Gestational age matched exactly in 85.2 % and date of delivery in 99.5 %.

**Conclusions:**

For selected medications used for chronic conditions, the agreement between self-reports and dispensing data was high. For medications with low to very low prevalence of use, PABAK provides a more reliable measure of agreement. Maternal reports and dispensing data are complementary to each other to increase the reliability of information on the use of medications during pregnancy. Birth certificates provide reliable data on the timing of pregnancy. FVG health databases are a valuable source of data for pregnancy research.

**Electronic supplementary material:**

The online version of this article (doi:10.1186/s12884-015-0745-3) contains supplementary material, which is available to authorized users.

## Background

Maternal use of prescription medications during pregnancy is common, with prevalence ranging from 27 to 99 % in developed countries [1]. In Italy, a prevalence of about 50 % has been reported [2].

Pregnant women are generally not included in pre-authorization studies, thus the risk–benefit profile of medicines used in pregnancy is assessed mostly through post-authorization studies. The assessment of the association between maternal use of medications during pregnancy and pregnancy or infant outcomes often rely on pregnancy medication exposure registries [3, 4] and on studies using administrative databases [5–7], registering prescriptions at the general physician prescription level or at pharmacy dispensing level.

Pregnancy registries can provide timely ascertainment of exposure and outcomes, and good quality information on their temporal association when data are collected prospectively. Limitations include: (a) potential for selection bias, as registration is spontaneous, (b) insufficient power for some outcomes, (c) problems in identifying an appropriate comparison group, (d) quality and completeness of information depends on healthcare providers and/or maternal reporting [8].

Administrative databases represent an efficient and cost-effective source of data on large populations, and they allow researchers to identify exposure, regardless of information on outcome [9]. However their use in assessing medication exposure has some limitations. In particular, prescription filling or redemption is a proxy for medication consumption. Noncompliance and medication borrowing or sharing [10] may lead to overestimation of use and exposure misclassification. It has been estimated that 6 % of dispensed medications were not used [11]. Moreover, information on the use of non-prescription and over- the- counter (OTC) medications, herbal preparations and medications taken in the hospital, is not captured.

Other approaches include case–control studies/surveillance, and cohort studies. In ad hoc studies, maternal self-reports have often been used to measure medication use in pregnancy. Inaccurate recall, susceptibility to bias and under-reporting are among the limitations of this tool. The accuracy of reporting has been shown to vary by therapeutic class [12], type of use (chronic vs. occasional) [13] and to depend on data collection methods and questionnaire design [14–16].

Due to the limitations of maternal self-reports and prescription databases, neither of these sources can be considered the ‘gold standard’ to assess the use of medications.

Several studies compared self-reported and database information on medication use [17–19], in specific subgroups, such as the elderly [20, 21], adolescents [22], hypertensive patients [16], or for specific medications or therapeutic classes, such as oral contraceptives [23], hormone replacement therapy [24], psychoactive medications [25], nonsteroidal anti-inflammatory drugs [26].

Few studies have been conducted in pregnant women, comparing maternal reports of medication use during pregnancy and database information [12, 14, 27–30]. In general, the results showed that medications taken for long courses or chronically, such as antidiabetic agents, medications for thyroid conditions and for asthma, antiepileptics and antihypertensives, had generally higher agreement than medications taken occasionally.

Another key methodological issue is the accuracy of information on the use of medications during pregnancy and on pregnancy timing. The latter is needed to assign etiologically relevant ‘time windows’ of exposure to medication at the exact gestational age.

In a cohort of 767 women, resident of Friuli Venezia Giulia (FVG) region, Northeast Italy, and recruited from 2007 to 2009 at the first visit in a prenatal clinic, we compared (a) self-reported information on medication use during pregnancy with data from the regional outpatient dispensing database; and (b) self-reported information on gestational age at birth and on date of delivery with data from the birth certificate database. Moreover, we assessed the effect of women characteristics on the likelihood of agreement.

## Methods

### Data sources

The sources of data were selected FVG health databases, recording computerized information on the use of health services for the residents of the region. All residents are registered with the Regional Health System, providing universal access to health care. A unique personal identifier links anonymized individual records. For this study, the outpatient dispensing and birth certificate databases were used.

The database used in this study records prescriptions at pharmacy redemption level. The database captures all redeemed prescriptions for reimbursed medications dispensed to residents of the region. Prescription medications are reimbursed to residents, including pregnant women.

For each redeemed prescription, the following information is recorded: date of redemption, active substance (description and Anatomical Therapeutic and Chemical ATC classification code [31]), brand, quantity, strength, dispensed form, number of units and number of refills. Information on the indication and the prescribed dosage regimen are not recorded.

The birth certificate database records data on all births in FVG since 1989. For each birth, the information recorded includes: gestational age at the first prenatal visit, at the first ultrasound examination and at delivery, date of delivery, number of prenatal visits and ultrasound examinations, gestational hypertension.

The Direzione Centrale Salute, Integrazione Socio Sanitaria e Politiche Sociali, Regione Friuli Venezia Giulia granted permission to access all above mentioned anonymized databases.

### Study cohort

Pregnant women attending their first prenatal visit (between 20 and 22 weeks of gestation) at the Institute for Maternal and Child Health - IRCCS “Burlo Garofolo”, in Trieste, FVG, from April 3, 2007 to March 3, 2009 were eligible to be included in this prospective cohort. Eligible women had to be resident in FVG for at least 2 years, in order to be covered by the regional health databases for a period of time before pregnancy, as another objective of this study was to assess the effect of maternal medication and behavioral exposures before pregnancy on the health of the mother and child. Moreover, women had to be fluent in Italian and at least 18 years old. Women with complicated or twin pregnancies were excluded.

Complicated pregnancies were defined as those with maternal abnormalities of the reproductive tract, uterine fibroids, pre-existing chronic illness such as cancer, AIDS, severe heart disease, severe kidney disease, severe Crohn’s disease or ulcerative colitis, and those with foetal congenital defects. A complicated pregnancy was determined at the time of recruitment. According to protocol, when a complication emerged in a prenatal examination (e.g. a prenatal tests indicated that the foetus had congenital defects), the woman was excluded from the study. However, no women were excluded for a complicated pregnancy, or any other reason, after recruitment. All eligible women recruited in the study were included in statistical analysis.

During the recruitment period, about 1800 live births per year were recorded in Trieste and 9000 in FVG [32].

### Data collection

Women who agreed to participate filled in a self-administered questionnaire between the 28th week of estimated gestational age and 1 month after delivery. The questionnaire inquired on the use of medications during the pregnancy. Women answering ‘Yes’ to the question ‘Have you ever taken medications – on a regular basis - during pregnancy?’ were asked to indicate the brand name and/or the name of the active substance and the indication. (‘Which medications have you used during pregnancy? Please list the commercial name of each medication, active substance, if known, and its indication’). In the instructions for completing the questions, ‘regular basis’ was defined as ‘the assumption of a medication for 4 or more times per week or for more than two weeks’.

Data on brand name, active substance and indication of up to six medications were collected. The questions were open-ended.

The questionnaire collected also information on women social and demographic characteristics (country of origin, age, level of education, marital status and profession), health behaviours and conditions (smoking, comorbidities before or during pregnancy, such as diabetes, asthma, allergy, epilepsy, hypertension, vomit, hypothyroidism, hyperthyroidism, lupus, rheumatic diseases, urinary infections, infections, fever, seizures, anemia, cardiovascular diseases, neurological diseases), prior pregnancies (gravidity), gestational age at birth and date of delivery. The date of questionnaire completion was also recorded. The questionnaire is provided as an Additional file 1.

For each woman, through record linkage using an individual identifier, we extracted from health databases the records of (a) prescriptions redeemed from 2006 to 2012 and (b) birth certificate. All prescriptions redeemed from the estimated date of conception to the date of delivery were considered during the pregnancy. The estimated date of conception was obtained by subtracting gestational age at birth from the date of delivery.

Because of the lack of a true gold standard, the agreement between questionnaire self-reports and prescriptions redemption data was evaluated by means of Kappa coefficient [33], with 95 % confidence interval (95 % CI) based on asymptotic standard error. Kappa values were interpreted according to Landis and Koch categorization [34] as almost perfect (>0.80), substantial (0.61-0.80), moderate (0.41-0.60), fair (0.21-0.40), slight (0.00-0.20) and poor (<0.00). Prevalence and bias indices and prevalence and bias-adjusted Kappa (PABAK) [35] were calculated. A SAS macro [36] was used for this analysis.

To help the interpretation of the Kappa values, we also calculated sensitivity, specificity, positive and negative predictive value, with 95 % confidence interval (95 % CI). The prescription database was the reference standard. Confidence intervals were calculated according to the method by Wilson [37] to avoid aberrations.

For women who completed the questionnaire before the delivery, the prescriptions dispensed from the estimated date of conception to the date of questionnaire completion were considered for the assessment of agreement.

The same statistics were calculated to assess the agreement between hypertension during pregnancy reported in questionnaire and recorded in the birth certificate database. Hypertension during pregnancy, both reported in questionnaire and recorded in the birth certificate database, was also compared with the use antihypertensive medications, both reported and recorded in the dispensing database.

The Odds Ratio (OR), with 95 % CI, of having at least one agreement between questionnaire and prescription database was calculated through unconditional logistic regression. The following variables were evaluated through uni- and multi-variable analysis: age at delivery, level of education, prior pregnancies, smoking status, comorbidities during pregnancy, country of origin, time of completion, marital status, number of visits and of ultrasound imaging during pregnancy, number of medications reported in questionnaire. The manual process of multivariate model building included entering individual terms and evaluating the likelihood ratio test for inclusion of each variable in the model. Only variables that explained the variability or modified the regression coefficient estimators were retained. The final model included age at delivery, level of education, prior pregnancies, smoking status, and comorbidities during pregnancy and country of origin. Women who did not report any medication use and without any prescription, were excluded from this analysis.

The percentage of women matching exactly or with ±1 and ±2 days of difference, on the date of delivery and gestational age at birth was calculated.

The statistical analysis was performed with SAS© software, version 9.3 (SAS, Cary, NC, USA).

### Ethics committee review

The study protocol was approved by the Ethics Committees at the University Hospital of Udine and at the Institute for Maternal and Child Health of Trieste. Written informed consent for participation in the study was obtained.

## Results

The cohort included 767 women of whom, 305 (39.8 %) reportedly used medications during pregnancy (Table 1). In this group, the percentages of women aged ≥35 years at delivery (41.2 % vs. 34.4 %), of primiparae (46.6 % vs. 44.5 %) and of never smokers (62.3 % vs. 53.3 %) were higher than those among nonusers. Users also reported more prenatal visits (≥8: 67.9 % vs. 64.2 %) and ultrasound examinations (≥5: 58.0 % vs. 54.3 %) than nonusers. Seven women did not complete the question on medication use. Only 5 (0.65 %) women reported 6 medications, 5 (0.65 %) reported 5 and 152 (19.8 %) reported the use of only 1 medication. The median number of medications reported was 1 (25°; 75° percentile: 1; 2) and the mean was 1.8 (standard deviation 1.09).

**Table 1:** Women characteristics by questionnaire self-reported use of medications

	Questionnaire-reported use of medications								
	Users		Non users		Not reported		Chi square p^a^	Total	
	(*N* = 305)		(*N* = 455)		(*N* = 7)			(*N* = 767)	
	*N*	%	*N*	%	*N*	%		*N*	%
Age at delivery (years)									
<25	20	6.6	22	4.8	0	-	0.1954	42	5.5
25-29	42	13.8	68	14.9	1	14.3		111	14.5
30-34	117	38.4	209	45.9	1	14.3		327	42.6
35-39	100	32.7	125	27.6	4	57.1		229	29.8
40+	26	8.5	31	6.8	1	14.3		58	7.6
Prior pregnancies^b^									
None	142	46.6	202	44.5	4	57.1	0.0182	348	45.4
1-2	131	42.9	226	49.8	2	28.6		359	46.8
3+	32	10.5	25	5.5	1	14.3		58	7.6
Marital status^c^									
Married	275	90.2	401	88.4	7	100.0	0.4642	683	89.0
Single	28	9.2	49	10.5	0	-		77	10.0
Level of education^c^									
< High school	48	15.7	88	19.4	3	42.9	0.1480	139	18.1
High school	140	45.9	222	48.8	2	28.6		364	47.5
University	116	38.0	144	31.7	2	28.6		262	34.2
Country of origin^c^									
Italy	279	91.5	415	91.4	7	100.0	0.9429	701	91.4
Other	24	7.9	35	7.7	0	-		59	7.8
Working status^c^									
Employed on maternity leave	225	73.8	338	74.3	5	71.4	0.7015	568	74.0
Currently employed	22	7.2	35	7.7	0	-		57	7.4
Housewife	26	8.5	39	8.6	2	28.6		67	8.7
Unemployed	20	6.6	42	9.2	0	-		62	8.1
Occupation									
Armed forces occupations	2	0.7	2	0.4	-	-	0.0044	4	0.5
Manager	16	5.2	13	2.9	-	-		29	3.8
Professional	16	5.2	51	11.2	-	-		67	8.7
Technicians and associate professionals	35	11.5	62	13.6	-	-		97	12.6
Clerical support workers	108	35.4	191	42.0	3	42.9		302	39.4
Service and sales workers	38	12.5	34	7.5	1	14.3		73	9.5
Craft and related trades workers	14	4.6	21	4.6	-	-		34	4.4
Plant and machine operators, and assemblers	10	3.3	11	2.4	-	-		21	2.7
Elementary occupations	23	7.5	17	3.7	-	-		40	5.2
Prenatal care visits (number)									
<7	50	16.4	98	21.5	3	42.9	0.2215	151	19.7
7	48	15.7	65	14.3	2	28.6		115	15.0
8	61	20.0	100	22.0	1	14.3		162	21.1
9 or more	146	47.9	192	42.2	1	14.3		339	44.2
Prenatal ultrasound imaging (number)									
<4	72	23.6	131	28.8	3	42.9	0.0425	206	26.9
4	56	18.4	77	16.9	1	14.3		134	17.5
5 to 7	79	25.9	139	30.6	2	28.6		220	28.7
8 or more	98	32.1	108	23.7	1	14.3		207	27.0
Smoking status^c^									
Never smoker	190	62.3	242	53.3	4	57.1	0.0557	436	56.8
Current smoker	25	8.2	47	10.3	1	14.3		73	9.5
Ex-smoker, quitted before pregnancy	64	20.9	112	24.7	1	14.3		177	23.1
Ex-smoker, quitted during or after pregnancy	21	6.9	49	10.8	1	14.3		71	9.3
Prescriptions redeemed during pregnancy									
Yes	243	79.7	285	62.6	5	71.4	<.0001	541	70.5
No	62	20.3	170	37.4	2	28.6		226	29.5

^a^ Comparing users and non users

^b^ Prior pregnancies refer to gravidity

^c^ The percentages may not sum to 100 % due to missing data

Overall, 70.5 % of women (*N* = 541) redeemed at least one prescription during the pregnancy. Only 2 women were dispensed more than 6 different medications (one 7 and one 9). The median number of dispensing was 2 (25°; 75° percentile: 1; 2), the mean was 1.8 (standard deviation 1.01). Folic acid (36.0 % of women reported the use and 29.0 % had at least one dispensing) and iron (26.2 % and 28.6 %) were the most frequently used medications (Table 2). A total of 146 women (19.2 %) were dispensed antibiotics and 96 (12.6 %) progestogens, but only 20 (2.6 %) and 19 (2.5 %), respectively, reported their use. Of note, 5 women were dispensed antidepressants and one methadone. The use of these medications was not reported.

**Table 2:** Number of women classified as users by questionnaire and prescription redemption database, by therapeutic class, Kappa coefficient (Kappa), with 95 % confidence interval, strength of agreement, Positive and Negative Agreement, Prevalence Index, Bias Index, Prevalence and Bias adjusted Kappa (PABAK)

		Users				Kappa	95 % CI^a^	Strength of Agreement^b^	Positive Agreement	Negative Agreement	Prevalence Index	Bias Index	PABAK
		Questionnaire		database									
Therapeutic class	ATC^c^	*N*	%	*N*	%								
*Alimentary tract and metabolism*													
*Medications for acid related disorders*	A02	27	3.6	66	8.7	0.17	0.06; 0.29	slight	0.21	0.95	−0.879	−0.051	0.81
*Antacids*	A02A	21	2.8	28	3.7	0.18	0.02; 0.33	slight	0.20	0.97	−0.935	−0.009	0.90
*Medications for peptic ulcer and gastro-oesophageal reflux*	A02B	7	0.9	42	5.5	0.15	0.01; 0.29	slight	0.16	0.97	−0.935	−0.046	0.89
*Medications for functional gastrointestinal disorders*	A03	12	1.6	0	-	0.00	- -	poor	0.00	0.99	−0.984	0.016	0.97
*Bile and liver therapy*	A05	2	0.3	3	0.4	0.40	−0.15; 0.94	fair	0.40	0.99	−0.993	−0.001	0.99
*Laxatives and antidiarrheals*	A06	4	0.5	1	0.1	0.00	−0.00; 0.00	poor	0.00	0.99	−0.993	0.004	0.99
*Insulin*	A10A	1	0.1	3	0.4	0.50	−0.10; 1.00	moderate	0.50	0.99	−0.995	−0.003	0.99
*Vitamins and mineral supplements*	A11, A12	18	2.4	6	0.8	0.00	−0.02; 0.00	poor	0.00	0.98	−0.968	0.016	0.94
*Blood and blood forming organs*													
*Antithrombotic agents*	B01	24	3.2	32	4.2	0.70	0.57; 0.84	substantial	0.71	0.99	−0.926	−0.010	0.96
*Heparins*	B01AB	14	1.8	23	3.0	0.64	0.46; 0.82	substantial	0.65	0.99	−0.951	−0.012	0.97
*Platelet aggregation inhibitors*	B01AC	14	1.8	12	1.6	0.76	0.58; 0.95	substantial	0.77	0.99	−0.966	0.003	0.98
*Antihemorrhagics*	B02	0	-	5	0.7	0.00	- -	poor	0.00	0.99	−0.993	−0.007	0.99
*Iron*	B03A	199	26.2	217	28.6	0.49	0.42; 0.56	moderate	0.63	0.86	−0.452	−0.024	0.59
*Folic acid*	B03B	273	36.0	220	29.0	0.11	0.04; 0.18	slight	0.40	0.71	−0.355	0.070	0.22
*Solutions*	B05BB	0	-	2	0.3	0.00	- -	poor	0.00	0.99	−0.99	−0.001	0.99
*Cardiovascular system*													
*Antihypertensive medications*	C02, C07, C08, C09A	6	0.8	8	1.0	0.86	0.66; 1.00	almost perfect	0.86	0.99	−0.98	−0.003	0.99
*Methyldopa*	C02	0	-	1	0.1	0.00	- -	poor	0.00	0.99	−0.99	−0.001	0.99
*Beta-blocking agents*	C07	3	0.4	3	0.4	1.00	1.00; 1.00	almost perfect	1.00	1.00	−0.99	0.000	1.00
*Calcium channel blockers*	C08	5	0.7	7	0.9	0.83	0.60; 1.00	almost perfect	0.83	0.99	−0.98	−0.003	0.99
*Ace inhibitors*	C09A	0	-	1	0.1	0.00	- -	poor	0.00	0.99	−0.99	−0.001	0.99
*Lipid modifying agents*	C10A	0	-	1	0.1	0.00	- -	poor	0.00	0.99	−0.99	−0.001	0.99
*Diuretics*	C03	0	-	2	0.3	0.00	- -	poor	0.00	0.99	−0.99	−0.003	0.99
*Vasoprotectives*	C05C	2	0.3	0	-	0.00	- -	poor	0.00	0.99	−0.99	0.003	0.99
*Genito-urinary system and sex hormones*													
*Gynaecological antiinfectives - antiseptics*	G01A	7	0.9	0	-	0.00	- -	poor	0.00	0.99	−0.991	0.009	0.98
*Sympathomimetics, labour repressants*	G02CA	10	1.3	1	0.1	0.18	−0.12; 0.48	slight	0.18	0.99	−0.985	0.012	0.98
*Prolactin inhibitors*	G02CB	0	-	3	0.4	0.00	- -	poor	0.00	0.99	−0.996	−0.004	0.99
*Hormonal contraceptives*	G03A	0	-	2	0.3	0.00	- -	poor	0.00	0.99	−0.997	−0.003	0.99
*Estrogens*	G03C	0	-	3	0.4	0.00	- -	poor	0.00	0.99	−0.996	0.004	0.99
*Progestogens*	G03D	19	2.5	96	12.6	0.28	0.18; 0.39	fair	0.31	0.94	−0.848	−0.104	0.79
*Gonadotrophins*	G03G	0	-	3	0.4	0.00	- -	poor	0.00	0.99	−0.996	−0.004	0.99
*Systemic hormonal preparations*													
*Glucocorticoid, systemic*	H02A	5	0.7	9	1.2	0.28	−0.03; 0.59	fair	0.29	0.99	−0.982	−0.005	0.97
*Thyroid preparations*	H03	35	4.6	39	5.1	0.86	0.77; 0.94	almost perfect	0.86	0.99	−0.902	−0.005	0.97
*Thyroid hormones*	H03A	33	4.3	39	5.1	0.88	0.80; 0.96	almost perfect	0.89	0.99	−0.905	−0.008	0.98
*Antithyroid preparations*	H03B	2	0.3	0	-	0.00	- -	poor	0.00	0.99	−0.997	0.003	0.99
*Anti-infective agents*													
*Antibiotics, systemic*	J01	20	2.6	146	19.2	0.12	0.05; 0.18	slight	0.16	0.90	−0.781	−0.166	0.63
*Antimycotics, systemic*	J02	1	0.1	4	0.5	0.40	−0.14; 0.94	fair	0.40	0.99	−0.993	−0.004	0.99
*Antivirals, systemic*	J05	1	0.1	5	0.7	0.33	−0.15; 0.82	fair	0.33	0.99	−0.992	−0.005	0.99
*Immune sera and immunoglobulins*	J06B	0	-	6	0.8	0.00	- -	poor	0.00	0.99	−0.992	−0.008	0.98
*Musculo-skeletal system*													
*Non-steroidal anti-inflammatory drugs*	M01A	2	0.3	11	1.4	0.00	−0.01; 0.00	poor	0.00	0.99	−0.983	−0.012	0.97
*Bisphosphonates*	M05B	0	-	1	0.1	0.00	- -	poor	0.00	0.99	−0.999	−0.001	0.99
*Nervous system*													
*Non-opioid analgesics*	N02BE	47	6.2	2	0.3	0.00	−0.01; 0.00	poor	0.00	0.97	−0.935	0.059	0.87
*Selective serotonin agonists*	N02CC	1	0.1	2	0.3	0.00	−0.00; 0.00	poor	0.00	0.99	−0.996	−0.001	0.99
*Antiepileptic medications*	N03	1	0.1	1	0.1	1.00	1.00; 1.00	almost perfect	1.00	1.00	−0.997	0.000	1.00
*Antidepressants*	N06A	0	-	5	0.7	0.00	- -	poor	0.00	0.99	−0.993	−0.007	0.99
*Methadone*	N07B	0	-	1	0.1	0.00	- -	poor	0.00	0.99	−0.999	−0.001	0.99
*Antiparasitic products*													
*Antiprotozoals and antinematodals*	P01	0	-	2	0.3	0.00	- -	poor	0.00	0.99	−0.997	−0.003	0.99
*Respiratory system*													
*Medications for obstructive airway disease*	R03	7	0.9	29	3.8	0.27	0.08; 0.46	fair	0.28	0.98	−0.953	−0.029	0.93
*Adrenergic inhalants*	R03A	5	0.7	11	1.4	0.49	0.19; 0.80	moderate	0.50	0.99	−0.980	−0.008	0.98
*Other inhalants*	R03B	1	0.1	22	2.9	0.08	−0.07; 0.24	slight	0.09	0.99	−0.970	−0.028	0.94
*Adrenergics, systemic*	R03CA	1	0.1	0	-	0.00	- -	poor	0.00	0.99	−0.999	0.001	0.99
*Nasal decongestants and other topical*	R01A	2	0.3	0	-	0.00	- -	poor	0.00	0.99	−0.997	0.003	0.99
*Cough and cold preparations*	R05	5	0.7	0	-	0.00	- -	poor	0.00	0.99	−0.993	0.007	0.99
*Antihistamines for systemic use*	R06A	3	0.4	5	0.7	0.50	0.07; 0.92	moderate	0.50	0.99	−0.990	−0.003	0.99

^a^ 95 % CI = 95 % Confidence Interval

^b^ According to Landis and Koch [22]

^c^ Anatomical Therapeutic and Chemical classification code [20]

Kappa and PABAK values indicated almost perfect to substantial agreement for antihypertensive medications (Kappa 0.86 and PABAK 0.99), thyroid hormones (0.88 and 0.98), antiepileptic medications (1.00 and 1.00), antithrombotic agents (0.70 and 0.96).

Except for iron (PABAK 0.59), all medications with Kappa 0.60 to 0.21, indicating moderate to fair agreement, had PABAK ≥0.79, e.g. insulin (Kappa 0.50 and PABAK 0.99), antihistamines for systemic use (0.50 and 0.99) and progestogens (0.28 and 0.79).

Except for folic acid (Kappa 0.11 and PABAK 0.22), PABAK was higher than Kappa when this latter indicated slight agreement, such as for antibiotics (0.12 and 0.63), labour repressants (0.18 and 0.98) and medications for acid related disorders (0.17 and 0.81). When Kappa indicated poor agreement, e.g. for non-steroidal anti-inflammatory drugs, non-opioid analgesics or selective serotonin agonists, PABAK was >0.80. The results did not vary when Kappa and PABAK were calculated separately according to the time of questionnaire completion (i.e. before or after the delivery) (Additional file 2: Table S1).

For all medications, the sensitivity of questionnaire vs. database was lower than specificity, and the negative predictive value was >0.90 with the exceptions of iron (0.84), folic acid (0.75), progestogens (0.89), antibiotics (0.82) (Table 3).

**Table 3:** Comparison of questionnaire to prescription redemption database, by therapeutic class, Sensitivity, Specificity, Positive Predictive Value, Negative Predictive Value, with 95 % confidence interval

Therapeutic class	ATC^a^	Sensitivity	95 % CI^b^		Specificity	95 % CI^b^	PPV^c^	95 % CI^b^		NPV^d^	95 % CI^b^
*Alimentary tract and metabolism*											
*Medications for acid related disorders*	A02	0.15	0.13; 0.18		0.98	0.96; 0.98	0.37	0.22; 0.56		0.92	0.90; 0.94
*Antacids*	A02A	0.18	0.15; 0.21		0.98	0.97; 0.99	0.24	0.11; 0.45		0.97	0.95; 0.98
*Medications for peptic ulcer and gastro-oesophageal reflux*	A02B	0.10	0.08; 0.12		1.00	0.99; 1.00	0.57	0.25; 0.84		0.95	0.93; 0.96
*Medications for functional gastrointestinal disorders*	A03	-	-	-	0.98	0.97; 0.99	0.00	0.00; 0.24		1.00	0.99; 1.00
*Bile and liver therapy*	A05	0.33	0.30; 0.37		1.00	0.99; 1.00	0.50	0.09; 0.91		1.00	0.99; 1.00
*Laxatives and antidiarrheals*	A06	0.00	-	-	0.99	0.99; 1.00	0.00	0.00; 0.49		1.00	0.99; 1.00
*Insulin*	A10A	0.33	0.30; 0.37		1.00	1.00; 1.00	1.00	0.21; 1.00		1.00	0.99; 1.00
*Vitamins and mineral supplements*	A11, A12	0.00	-	-	0.98	0.96; 0.99	0.00	0.00; 0.18		0.99	0.98; 1.00
*Blood and blood forming organs*											
*Antithrombotic agents*	B01	0.61	0.57; 0.64		0.99	0.99; 1.00	0.83	0.64; 0.93		0.98	0.97; 0.99
*Heparins*	B01AB	0.50	0.46; 0.54		1.00	0.99; 1.00	0.86	0.60; 0.96		0.98	0.97; 0.99
*Platelet aggregation inhibitors*	B01AC	0.83	0.81; 0.86		0.99	0.99; 1.00	0.71	0.45; 0.88		1.00	0.99; 1.00
*Antihemorrhagics*	B02	0.00	-	-	1.00	0.99; 1.00	-	-	-	0.99	0.98; 1.00
*Iron*	B03A	0.60	0.56; 0.63		0.88	0.85; 0.90	0.66	0.59; 0.72		0.84	0.81; 0.87
*Folic acid*	B03B	0.44	0.41; 0.48		0.68	0.65; 0.71	0.36	0.30; 0.42		0.75	0.71; 0.79
*Solutions*	B05BB	0.00	-	-	1.00	1.00; 1.00	-	-	-	1.00	0.99; 1.00
*Cardiovascular system*											
*Antihypertensive medications*	C02, C07, C08, C09A	0.75	0.72; 0.78		1.00	1.00; 1.00	1.00	0.61; 1.00		1.00	0.99; 1.00
*Methyldopa*	C02	0.00	-	-	1.00	1.00; 1.00	-	-	-	1.00	0.99; 1.00
*Beta-blocking agents*	C07	1.00	1.00; 1.00		1.00	1.00; 1.00	1.00	0.44; 1.00		1.00	0.99; 1.00
*Calcium channel blockers*	C08	0.71	0.68; 0.75		1.00	1.00; 1.00	1.00	0.57; 1.00		1.00	0.99; 1.00
*Ace inhibitors*	C09A	0.00	-	-	1.00	1.00; 1.00	-	-	-	1.00	0.99; 1.00
*Lipid modifying agents*	C10A	0.00	-	-	1.00	1.00; 1.00	-	-	-	1.00	0.99; 1.00
*Diuretics*	C03	0.00	-	-	1.00	1.00; 1.00	-	-	-	1.00	0.99; 1.00
*Vasoprotectives*	C05C	-	-	-	1.00	0.99; 1.00	0.00	0.00; 0.66		1.00	1.00; 1.00
*Genito urinary system and sex hormones*											
*Gynaecological antiinfectives and antiseptics*	G01A	-	-	-	0.99	0.98; 1.00	0.00	0.00; 0.35		1.00	0.99; 1.00
*Sympathomimetics, labour repressants*	G02CA	1.00	1.00; 1.00		0.99	0.98; 0.99	0.10	0.02; 0.40		1.00	0.99; 1.00
*Prolactin inhibitors*	G02CB	0.00	-	-	1.00	1.00; 1.00	-	-	-	1.00	0.99; 1.00
*Hormonal contraceptives*	G03A	0.00	-	-	1.00	1.00; 1.00	-	-	-	1.00	0.99; 1.00
*Estrogens*	G03C	0.00	-	-	1.00	1.00; 1.00	-	-	-	1.00	0.99; 1.00
*Progestogens*	G03D	0.17	0.15; 0.20		1.00	0.99; 1.00	0.94	0.74; 0.99		0.89	0.87; 0.91
*Gonadotrophins*	G03G	0.00	-	-	1.00	1.00; 1.00	-	-	-	1.00	0.99; 1.00
*Systemic hormonal preparations*											
*Glucocorticoid, systemic*	H02A	0.22	0.19; 0.25		1.00	0.99; 1.00	0.40	0.12; 0.77		0.99	0.98; 1.00
*Thyroid preparations*	H03	0.82	0.79; 0.85		1.00	0.99; 1.00	0.91	0.78; 0.97		0.99	0.98; 1.00
*Thyroid hormones*	H03A	0.82	0.79; 0.85		1.00	0.99; 1.00	0.97	0.85; 0.99		0.99	0.98; 1.00
*Antithyroid preparations*	H03B	-	-	-	1.00	0.99; 1.00	0.00	0.00; 0.66		1.00	1.00; 1.00
*Anti-infective agents*											
*Antibiotics, systemic*	J01	0.09	0.07; 0.11		0.99	0.98; 0.99	0.65	0.43; 0.82		0.82	0.79; 0.85
*Antimycotics, systemic*	J02	0.25	0.22; 0.28		1.00	1.00; 1.00	1.00	0.21; 1.00		1.00	0.99; 1.00
*Antivirals, systemic*	J05	0.20	0.17; 0.23		1.00	1.00; 1.00	1.00	0.21; 1.00		0.99	0.99; 1.00
*Immune sera and immunoglobulins*	J06B	0.00	-	-	1.00	1.00; 1.00	-	-	-	0.99	0.98; 1.00
*Musculo-skeletal system*											
*Non-steroidal anti-inflammatory drugs*	M01A	0.00	-	-	1.00	0.99; 1.00	0.00	0.00; 0.66		0.99	0.97; 0.99
*Bisphosphonates*	M05B	0.00	-	-	1.00	1.00; 1.00	-	-	-	1.00	0.99; 1.00
*Nervous system*											
*Non-opioid analgesics*	N02BE	0.00	-	-	0.94	0.92; 0.95	0.00	0.00; 0.08		1.00	0.99; 1.00
*Selective serotonin agonists*	N02CC	0.00	-	-	1.00	0.99; 1.00	0.00	0.00; 0.79		1.00	0.99; 1.00
*Antiepileptic medications*	N03	1.00	1.00; 1.00		1.00	1.00; 1.00	1.00	0.21; 1.00		1.00	1.00; 1.00
*Antidepressants*	N06A	0.00	-	-	1.00	1.00; 1.00	-	-	-	0.99	0.98; 1.00
*Methadone*	N07B	0.00	-	-	1.00	1.00; 1.00	-	-	-	1.00	0.99; 1.00
*Antiparasitic products*											
*Antiprotozoals and antinematodals*	P01	0.00	-	-	-	1.00; 1.00	-	-	-	1.00	0.99; 1.00
*Respiratory system*											
*Medications for obstructive airway disease*	R03	0.17	0.14; 0.19		1.00	0.99; 1.00	0.83	0.44; 0.97		0.97	0.95; 0.98
*Adrenergic, inhalants*	R03A	0.36	0.33; 0.40		1.00	1.00; 1.00	1.00	0.51; 1.00		0.99	0.98; 1.00
*Other inhalants*	R03B	0.04	0.03; 0.06		1.00	1.00; 1.00	1.00	0.21; 1.00		0.97	0.96; 0.98
*Adrenergics, systemic*	R03CA	-	-	-	1.00	0.99; 1.00	0.00	0.00; 0.79		1.00	1.00; 1.00
*Nasal decongestants and other topical*	R01A	-	-	-	1.00	0.99; 1.00	0.00	0.00; 0.66		1.00	1.00; 1.00
*Cough and cold preparations*	R05	-	-	-	0.99	0.98; 1.00	0.00	0.00; 0.43		1.00	0.99; 1.00
*Antihistamines for systemic use*	R06A	0.40	0.37; 0.44		1.00	0.99; 1.00	0.67	0.21; 0.94		1.00	0.99; 1.00

^a^Anatomical Therapeutic and Chemical ATC classification code [20]

^b^95 % CI = 95 % Confidence Interval

^c^PPV = Positive Predictive Value

^d^NPV = Negative Predictive Value

When simultaneously adjusted for age at delivery, level of education, prior pregnancies, smoking status, comorbidities during pregnancy and country of origin, the OR of ≥1 agreement was 0.88 (95 % CI 0.46- 1.67) in immigrant vs. Italy-born women, 0.48 (95 % CI 0.26- 0.90) in ex-smokers having quit during or after the pregnancy and 0.66 (95 % CI 0.36- 1.21) in current vs. never smokers (Table 4). The OR was 0.75 (95 % CI 0.53- 1.09) and 0.64 (95 % CI 0.38- 1.08) in women with high school and < high school, respectively, vs. those with a university degree.

**Table 4:** Odds ratio, with 95 % confidence interval, of having at least one agreement between questionnaire and prescription redemption database

		Agreement between questionnaire and database^a^									
		At least 1 (*N* = 266)		None (*N* = 375)		Univariate		Multivariate^a^		Multivariate^b^	
		*n*	%	*n*	%	Odds Ratio	95 % CI^b^	Odds Ratio	95 % CI^b^	Odds Ratio	95 % CI^b^
Age at delivery (years)											
	<25	20	7.6	18	4.8	1.71	0.87; 3.39	2.25	1.06; 4.79	2.11	0.90; 4.93
	25-29	27	10.1	61	16.3	0.68	0.41; 1.14	0.67	0.39; 1.14	0.57	0.31; 1.04
	30-34^c^	110	41.3	170	45.3	1.00	- -	1.00	- -	1.00	- -
	35-39	81	30.4	106	28.3	1.18	0.81; 1.72	1.33	0.90; 1.97	0.97	0.62; 1.52
	40+	28	10.6	20	5.3	2.16	1.16; 4.03	2.25	1.19; 4.26	2.34	1.15; 4.76
Country of origin^a^											
	Italy^c^	245	92.1	341	90.9	1.00	- -	1.00	- -	1.00	- -
	Other	19	7.1	30	8.0	0.88	0.48; 1.60	0.88	0.46; 1.67	0.83	0.30; 1.63
Level of education											
	< High school	39	14.7	71	19.0	0.59	0.37; 0.95	0.64	0.38; 1.08	0.64	0.35; 1.19
	High school	118	44.4	186	49.7	0.69	0.48; 0.97	0.75	0.53; 1.09	0.89	0.59; 1.35
	University^c^	108	40.6	117	31.3	1.00	- -	1.00	- -	1.00	- -
Smoking status^a^											
	Ex-smoker, having quit during or after pregnancy	17	6.4	41	10.9	0.50	0.27; 0.91	0.48	0.26; 0.90	0.61	0.30; 1.24
	Ex-smoker, having quit before pregnancy	56	21.2	80	21.3	0.85	0.57; 1.26	0.85	0.56; 1.29	0.91	0.57; 1.45
	Current smoker	19	7.2	40	10.7	0.57	0.32; 1.03	0.66	0.36; 1.21	0.78	0.39; 1.56
	Never smoker^c^	172	65.1	208	55.5	1.00	- -	1.00	- -	1.00	- -
Prior pregnancies											
	None	138	51.9	165	44.0	1.37	1.00; 1.88	1.50	1.07; 2.11	1.52	1.03; 2.24
	At least one^c^	128	48.1	210	56.0	1.00	- -	1.00	- -	1.00	- -
Comorbidities during pregnancy^d^											
	At least one	202	75.9	286	76.3	1.79	1.25; 2.55	1.55	1.01; 2.37	1.10	0.66; 1.81
	None^c^	61	22.9	89	23.7	1.00	- -	1.00	- -	1.00	- -
Number of medications reported in questionnaire											
	0	78	29.3	269	71.7	0.26	0.17; 0.39	- -	- -	0.26	0.17; 0.41
	1^c^	80	30.1	72	19.2	1.00	- -	- -	- -	1.00	- -
	2	65	24.4	23	6.1	2.54	1.43; 4.51	- -	- -	2.53	1.39; 4.59
	3 or more	43	16.2	11	2.9	3.52	1.69; 7.34	- -	- -	3.81	1.74; 8.34
Marital status											
	Married^c^	242	91.0	332	88.5	1.00	- -	- -	- -	- -	- -
	Single	23	8.6	38	10.1	0.83	0.48; 1.43	- -	- -	- -	- -
Time of questionnaire completion											
	Pre-delivery	127	47.7	204	54.4	0.77	0.56; 1.05	- -	- -	- -	- -
	Post-delivery^c^	139	52.3	171	45.6	1.00	- -	- -	- -	- -	- -
Prenatal care visits (number)											
	<7	47	17.7	75	20.0	0.89	0.54; 1.47	- -	- -	- -	- -
	7	41	15.4	57	15.2	0.96	0.60; 1.52	- -	- -	- -	- -
	8	52	19.5	75	20.0	0.92	0.61; 1.41	- -	- -	- -	- -
	9 + ^c^	126	47.4	168	44.8	1.00	- -	- -	- -	- -	- -
Ultrasound imaging during pregnancy (number)											
	<4	66	24.8	98	26.1	0.87	0.56; 1.33	- -	- -	- -	- -
	4	50	18.8	60	16.0	1.07	0.66; 1.73	- -	- -	- -	- -
	5-7	73	27.5	118	31.5	0.79	0.52; 1.21	- -	- -	- -	- -
	8 + ^c^	77	28.9	99	26.4	1.00	- -	- -	- -	- -	- -

^a^ Adjusted by age at delivery, level of education, prior pregnancies, smoking status, comorbidities during pregnancy, country of origin

^b^ 95 % CI = 95 % confidence interval

^c^ Reference category

^d^ It includes the following comorbidities occurring only during the pregnancy or before and during the pregnancy: diabetes, asthma, allergy, epilepsy, hypertension, vomit, hypothyroidism, hyperthyroidism, lupus, rheumatic diseases, urinary infections, infections, fever, seizures, anaemia, cardiovascular diseases, and neurological diseases

Women experiencing comorbidities during the pregnancy (OR 1.55, 95 % CI 1.01- 2.37), primiparae (OR 1.50, 95 % CI 1.07- 2.11) and those aged 40+ years (OR 2.25, 95 % CI 1.19- 4.26) vs. those aged 30–34 years, were significantly more likely to have ≥1agreement. The OR was also increased in women aged <25 (OR 2.25, 95 % CI 1.06- 4.79) and 35 to 39 years (OR 1.33, 95 % CI 0.90- 1.97).

Agreement was more likely with an increasing number of medications reported: compared to women reporting the use of 1 medication during pregnancy, the OR of having at least one agreement was 2.53 (95 % CI 1.39; 4.59) in those reporting 2, and 3.81 (95 % CI 1.74; 8.34) in those reporting 3 or more medications.

Gestational age matched exactly in 85.2 % of women (±1 or 2 days in 14.6 %) and date of delivery in 99.5 % (±1 day in 0.5 %) (Table 5). For number of prenatal visits and number of ultrasound examinations, concordance (±1) was 54.7 % and 57.2 %, respectively.

**Table 5:** Agreement between self-reported questionnaire and birth certificate database information

	Gestational age (weeks)		Date of delivery^a^ (day)		Prenatal visits (number)		Prenatal ultrasound examinations (number)	
	*N*	%	*N*	%	*N*	%	*N*	%
Exact match	634	85.2	759	99.5	186	24.2	254	33.1
±1	100	13.4	4	0.5	234	30.5	185	24.1
±2	9	1.2	-	-	106	13.8	89	11.6
±3+	1	0.1			241	31.4	239	31.2

^a^ The date of delivery was missing in the questionnaire for 4 women

Hypertension during pregnancy was reported by 33 (4.3 %) women but only 15 had this condition recorded in the birth certificate database (Kappa 0.40; 95 % CI 0.22-0.58; PABAK 0.92). Dispensing for antihypertensive medications had a positive predictive value (PPV) of 100 % for both self-reported and recorded hypertension and a negative predictive value (NPV) of 96.4 % (95 % CI 95.0-97.8) and 98.9 % (95 % CI 98.2-99.6), respectively (Additional file 1: Tables S2-S3).

## Discussion

About 40 % of women reported the use of medications and about 70 % redeemed at least one prescription during pregnancy. The agreement between self-reported data and database information varied greatly by therapeutic class. It was almost perfect to substantial for medications taken for chronic conditions, such as antihypertensive medications, thyroid hormones, antiepileptic and antithrombotic medications, while it was moderate to slight for OTC medications, such as iron and folic acid. These results are consistent with prior studies [12, 28–30]. Medications such as antibiotics or antivirals, taken occasionally, showed slight to fair Kappa-based agreement but, when prevalence and bias were taken into account, the agreement was higher. A prior study found high agreement for antibiotics [38]. The Kappa coefficient is influenced by the prevalence of the condition and by bias. Its value, therefore, was interpreted in the light of additional indices of agreement, such as PABAK. For several medications showing moderate to poor agreement, such as agents for obstructive airways disease and for acid related disorders, progestogens, labour repressants, non-opioid analgesics and antidepressants, the value of these indices suggested that the low value of Kappa was influenced by the low to very low prevalence of use in the population.

Several reasons may explain the level of agreement between self-reported data and prescription redemption records. The type of use affects the accuracy of recall, thus women may recall more accurately medications taken chronically or over longer periods than those taken occasionally. Questionnaire design and question structure influence recall [15, 16, 39]. Questions specific for individual medications/therapeutic classes or for indication, increase the percentage of affirmative answers [39]; memory aids increase the accuracy of reporting. In this study, the questionnaire was self-administered, questions were open and no memory aid was used. This limitation may have contributed to decrease the positive agreement, in particular for medications taken occasionally.

Antidepressants and methadone were prescribed but not reported. In a recent study, use of antidepressants was not reported by 22 % of users during the first trimester and by 38 % during the second and third [40].

Noncompliance and prescription medication borrowing or sharing [10] may also partially explain disagreement.

We did not consider the prescriptions redeemed before the conception date. However, women may have redeemed a prescription before and taken the medication also after conception, partly explaining the discrepancies between sources.

The database does not capture information on the use of OTC, non-prescription or non-reimbursed medications and herbal preparations. However, their use may have been reported in questionnaires, thus contributing to discrepancy between sources. The estimated prevalence of OTC use by pregnant women is not negligible. In the Netherlands, 12.5 % of pregnant women used OTC medications [41]. In the USA, OTC acetaminophen, ibuprofen, and pseudoephedrine were used by at least 65 %, 18 %, and 15 % of pregnant women, respectively [42].

Moreover, women may report medications taken in the hospital setting, not captured by prescription databases. The result for progestogens can be partly explained by in-hospital use, e.g. for the risk of abortion.

In prior studies, the recall of medications taken during the pregnancy was lower when assessed post-delivery vs. pre-delivery [43, 44]. The recall time span was several months to eight years. In our cohort, the agreement did not vary according to the time of questionnaire completion. The recall time span in our study was much shorter, median 30 days (25°-75° percentile 21–45 days). This result confirms that the recall of medication use during pregnancy is higher when data is collected shortly after the delivery.

Women sociodemographic characteristics, health behaviours and conditions influenced the probability of agreement. The agreement was less likely in immigrant women, those with less than university education and current or ex-smokers. Recall accuracy has been positively associated to maternal education [13, 44] and negatively to smoking during pregnancy [13, 29]. Smoking during pregnancy has been positively associated with poor attention for health, for instance women smokers more frequently do not take folic supplementation [45] and have low adherence to psychotropic medications [46]. Smokers may therefore have a less accurate recall of medications assumption in pregnancy.

In our study, agreement was more likely in primiparae, women experiencing comorbidities during pregnancy and those in the extreme age classes. Women at their first pregnancy, with poorer health condition or aged 40 years or older, may be more concerned on the pregnancy outcome and have a more accurate recall. Another study found that the recall certainty of dates of analgesic use in pregnancy was positively associated with maternal age [13].

The use of medications outside the coverage of the dispensing database, such as herbal medications or vitamin supplements, may be more frequent in subgroups of immigrant and young adult women. This differential use of medications not covered by the database may partially explain the lower likelihood of agreement in immigrant women and in those aged 25 to 29 years.

The likelihood of agreement increases with increasing number of medications reported. Women who use more medications may be those with health problems in pregnancy; therefore, they may recall better the medications used during it. The total number of medications has previously been positively associated with the recall accuracy of prescription analgesics use [13].

Databases do not always capture information on gestational age and date of delivery. Thus, the timing of exposure relative to pregnancy cannot be evaluated. This limitation hinders the use of databases for pregnancy research. The accurate timing of pregnancy is of great relevance for epidemiologic studies of exposures during pregnancy, including medications, and maternal or foetal and infant outcomes. We found a very high agreement for gestational age and date of delivery between questionnaire data and birth certificate records. This result is in line with a prior study, reporting a high agreement, with positive predictive value >90 %, between birth certificate and medical record data for gestational age [47].

The additional value of this study to the existing literature on the agreement between self-reports and database information on medication use during pregnancy includes the followings: (a) it was performed in a cohort with information on women demographic and socioeconomic characteristics, and it could, therefore, assess the factors associated with agreement; (b) in measuring agreement, the prevalence of the medication use was taken into account through the PABAK calculation; (c) the study evaluated in the same cohort both the agreement for medication use and for gestational age and date of delivery - the latter being crucial for evaluating the reliability of data on the timing of pregnancy.

A strength of this study is that all the women in the cohort were linked to dispensing and birth certificate records, without omissions of specific population subgroups (e.g. low socioeconomic level or immigrant status), confirming the high quality of FVG databases.

## Conclusions

The agreement between self-reports and prescription redemption data was high to very high for medications used for chronic conditions. Our findings confirm that maternal reports and prescription redemption data are complementary to each other to increase the reliability of information on the use of medications during pregnancy. Future studies using large administrative data should be considered to assess exposure also with a self-reported questionnaire in a subsample as an internal validation study. The results of this validation study could be used, e.g. in sensitivity analysis, to take into account the impact of possible exposure misclassification, on the association with the outcome.

To assess the use of medications not captured by database, such as OTC, herbal preparations, medications not reimbursed or used in the hospital setting, other sources should be considered, such as primary care or hospital electronic medical records.

The method choice of interview and questionnaire design should account for maternal factors affecting recall, such as sociodemographic and health behaviours, in the target population.

We found a very high agreement for gestational age and date of delivery between maternal reports and birth certificate database. This result suggests that birth certificates provide reliable data on the timing of pregnancy.

Our results show that FVG health databases are a valuable source of data for pregnancy research and for studies on the safety of medications during pregnancy.

## Additional files

Additional file 1:
**Study questionnaire.** (DOCX 24 kb) Additional file 2:
**Supplemental tables. Table S1.** Agreement between questionnaire and prescription redemption database for selected therapeutic classes by time of questionnaire completion. **Table S2.** Number of women with information on hypertension during pregnancy and agreement between questionnaire and birth certificate database. **Table S3.** Number of women with information on hypertension during pregnancy in questionnaire and in birth certificate database and use of antihypertensive medications according to questionnaire and prescription database. Positive Predictive Value and Negative Predictive Value of prescriptions for antihypertensive medications recorded in questionnaire and in birth certificate database. (DOCX 23 kb)

## References

[1] Daw JR, Hanley GE, Greyson DL, Morgan SG (2011). Prescription drug use during pregnancy in developed countries: a systematic review. Pharmacoepidemiol Drug Saf.

[2] Gagne JJ, Maio V, Berghella V, Louis DZ, Gonnella JS (2008). Prescription drug use during pregnancy: a population-based study in Regione Emilia-Romagna, Italy. Eur J Clin Pharmacol.

[3] Howard TB, Tassinari MS, Feibus KB, Mathis LL (2011). Monitoring for teratogenic signals: pregnancy registries and surveillance methods. Am J Med Genet C: Semin Med Genet.

[4] Artama M, Gissler M, Malm H, Ritvanen A (2011). Nationwide register-based surveillance system on drugs and pregnancy in Finland 1996–2006. Pharmacoepidemiol Drug Saf.

[5] Andrade SE, Raebel MA, Morse AN, Davis RL, Chan KA, Finkelstein JA (2006). Use of prescription medications with a potential for fetal harm among pregnant women. Pharmacoepidemiol Drug Saf.

[6] Colvin L, Slack-Smith L, Stanley FJ, Bower C (2010). Linking a pharmaceutical claims database with a birth defects registry to investigate birth defect rates of suspected teratogens. Pharmacoepidemiol Drug Saf.

[7] Charlton RA, Neville AJ, Jordan S, Pierini A, Damase-Michel C, Klungsoyr K (2014). Healthcare databases in Europe for studying medicine use and safety during pregnancy. Pharmacoepidemiol Drug Saf.

[8] Chambers CD, Andrews EB. Drug safety in Pregnancy. In: Mann R, Andrews EB, editors. Pharmacovigilance*.* 2nd ed. Chichester, England: John Wiley & Sons; 2007.

[9] Mitchell AA. Studies of Drug-induced Birth Defects**.** In: Strom BL, editor. Pharmacoepidemiology. 4th ed. Chichester, England: John Wiley & Sons; 2005.

[10] Petersen EE, Rasmussen SA, Daniel KL, Yazdy MM, Honein MA (2008). Prescription medication borrowing and sharing among women of reproductive age. J Womens Health (Larchmt).

[11] De Jong van den Berg LT, Feenstra N, Sorensen HT, Cornel MC (1999). Improvement of drug exposure data in a registration of congenital anomalies. Pilot-study: pharmacist and mother as sources for drug exposure data during pregnancy. EuroMAP Group. Europen Medicine and Pregnancy Group. Teratology.

[12] Olesen C, Sondergaard C, Thrane N, Nielsen GL, de Jong-van den Berg L, Olsen J (2001). Do pregnant women report use of dispensed medications?. Epidemiology.

[13] Radin RG, Mitchell AA, Werler MM (2013). Predictors of recall certainty of dates of analgesic medication use in pregnancy. Pharmacoepidemiol Drug Saf.

[14] de Jong-van den Berg LT, Waardenburg CM, Haaijer-Ruskamp FM, Dukes MN, Wesseling H (1993). Drug use in pregnancy: a comparative appraisal of data collecting methods. Eur J Clin Pharmacol.

[15] Gama H, Correia S, Lunet N (2009). Questionnaire design and the recall of pharmacological treatments: a systematic review. Pharmacoepidemiol Drug Saf.

[16] Klungel OH, de Boer A, Paes AH, Herings RM, Seidell JC, Bakker A (2000). Influence of question structure on the recall of self-reported drug use. J Clin Epidemiol.

[17] Nielsen MW, Sondergaard B, Kjoller M, Hansen EH (2008). Agreement between self-reported data on medicine use and prescription records vary according to method of analysis and therapeutic group. J Clin Epidemiol.

[18] Caskie GI, Willis SL, Warner Schaie K, Zanjani FA (2006). Congruence of medication information from a brown bag data collection and pharmacy records: findings from the Seattle longitudinal study. Exp Aging Res.

[19] Monster TB, Janssen WM, de Jong PE, de Jong-van den Berg LT (2002). Pharmacy data in epidemiological studies: an easy to obtain and reliable tool. Pharmacoepidemiol Drug Saf.

[20] Noize P, Bazin F, Dufouil C, Lechevallier-Michel N, Ancelin ML, Dartigues JF (2009). Comparison of health insurance claims and patient interviews in assessing drug use: data from the Three-City (3C) Study. Pharmacoepidemiol Drug Saf.

[21] Boudreau DM, Doescher MP, Saver BG, Jackson JE, Fishman PA (2005). Reliability of Group Health Cooperative automated pharmacy data by drug benefit status. Pharmacoepidemiol Drug Saf.

[22] Skurtveit S, Selmer R, Tverdal A, Furu K (2008). The validity of self-reported prescription medication use among adolescents varied by therapeutic class. J Clin Epidemiol.

[23] Norell SE, Boethius G, Persson I (1998). Oral contraceptive use: interview data versus pharmacy records. Int J Epidemiol.

[24] Strom BL, Schinnar R (2004). An interview strategy was critical for obtaining valid information on the use of hormone replacement therapy. J Clin Epidemiol.

[25] Haapea M, Miettunen J, Lindeman S, Joukamaa M, Koponen H (2010). Agreement between self-reported and pharmacy data on medication use in the Northern Finland 1966 Birth Cohort. Int J Methods Psychiatr Res.

[26] Grimaldi-Bensouda L, Rossignol M, Aubrun E, Benichou J, Abenhaim L (2012). Agreement between patients’ self-report and physicians’ prescriptions on nonsteroidal anti-inflammatory drugs and other drugs used in musculoskeletal disorders: the international Pharmacoepidemiologic General Research eXtension database. Pharmacoepidemiol Drug Saf.

[27] Bryant HE, Visser N, Love EJ (1989). Records, recall loss, and recall bias in pregnancy: a comparison of interview and medical records data of pregnant and postnatal women. Am J Public Health.

[28] Stephansson O, Granath F, Svensson T, Haglund B, Ekbom A, Kieler H (2011). Drug use during pregnancy in Sweden - assessed by the Prescribed Drug Register and the Medical Birth Register. Clin Epidemiol.

[29] van Gelder MM, van Rooij IA, de Walle HE, Roeleveld N, Bakker MK (2013). Maternal recall of prescription medication use during pregnancy using a paper-based questionnaire: a validation study in the Netherlands. Drug Saf.

[30] Sarangarm P, Young B, Rayburn W, Jaiswal P, Dodd M, Phelan S (2012). Agreement between self-report and prescription data in medical records for pregnant women. Birth Defects Res A Clin Mol Teratol.

[31] WHO Collaborating Centre for Drug Statistics Methodology NIoPH: ATC/DDD Index 2015. http://www.whocc.no/atc_ddd_index/ (last accessed on 28 November, 2014). In*.*

[32] ISTAT Italian Statistical Institute http://www.istat.it/it/files/2012/01/indicatoridemografici2011.pdf (last accessed 21, December 2014)*.*

[33] Thompson WD, Walter SD (1988). A reappraisal of the kappa coefficient. J Clin Epidemiol.

[34] Landis JR, Koch GG (1977). The measurement of observer agreement for categorical data. Biometrics.

[35] Byrt T, Bishop J, Carlin JB (1993). Bias, prevalence and kappa. J Clin Epidemiol.

[36] Cunningham M: More than Just the Kappa Coefficient: A Program to Fully Characterize Inter-Rater Reliability between Two Raters. Paper 242–2009. SAS Global Forum 2009. http://support.sas.com/resources/papers/proceedings09/242-2009.pdf [last accessed March, 17 2015].

[37] Newcombe RG (1998). Two-sided confidence intervals for the single proportion: comparison of seven methods. Stat Med.

[38] Espnes MG, Bjorge T, Engeland A (2011). Comparison of recorded medication use in the Medical Birth Registry of Norway with prescribed medicines registered in the Norwegian Prescription Database. Pharmacoepidemiol Drug Saf.

[39] Mitchell AA, Cottler LB, Shapiro S (1986). Effect of questionnaire design on recall of drug exposure in pregnancy. Am J Epidemiol.

[40] Kallen B, Nilsson E, Olausson PO (2011). Antidepressant use during pregnancy: comparison of data obtained from a prescription register and from antenatal care records. Eur J Clin Pharmacol.

[41] Verstappen GM, Smolders EJ, Munster JM, Aarnoudse JG, Hak E (2013). Prevalence and predictors of over-the-counter medication use among pregnant women: a cross-sectional study in the Netherlands. BMC Public Health.

[42] Werler MM, Mitchell AA, Hernandez-Diaz S, Honein MA (2005). Use of over-the-counter medications during pregnancy. Am J Obstet Gynecol.

[43] Feldman Y, Koren G, Mattice K, Shear H, Pellegrini E, MacLeod SM (1989). Determinants of recall and recall bias in studying drug and chemical exposure in pregnancy. Teratology.

[44] de Jong PCMP, Berns MPH, van Duynhoven YTHP, Nijdam WS, Eskes TKAB, Zielhuis GA (1995). Recall of medication during pregnancy: Validity and accuracy of an adjusted questionnaire. Pharmacoepidemiol Drug Saf.

[45] Baron R, Mannien J, de Jonge A, Heymans MW, Klomp T, Hutton EK (2013). Socio-demographic and lifestyle-related characteristics associated with self-reported any, daily and occasional smoking during pregnancy. PLoS One.

[46] Lupattelli A, Spigset O, Bjornsdottir I, Hameen-Anttila K, Mardby AC, Panchaud A (2015). Patterns and factors associated with low adherence to psychotropic medications during pregnancy—a cross-sectional, multinational web-based study. Depress Anxiety.

[47] Andrade SE, Scott PE, Davis RL, Li DK, Getahun D, Cheetham TC (2013). Validity of health plan and birth certificate data for pregnancy research. Pharmacoepidemiol Drug Saf.

