# Supplementary material for: Medication use during pregnancy, gestational age and date of delivery: agreement between maternal self-reports and health database information in a cohort
Source: BMC Pregnancy Childbirth. 2015 Nov 25;15:310. doi: 10.1186/s12884-015-0745-3 (PMC4660837; doi:10.1186/s12884-015-0745-3)
Supplement: Additional file 1: — Study questionnaire. (DOCX 24 kb) [file 12884_2015_745_MOESM1_ESM.docx]

Questionnaire code | _ | _ | _ | _ | _ | _ | *

Start of questionnaire completion dd |_|_| mm |_|_| yyyy |_|_|_|_|

**Sociodemographic data of the woman**

1. Date of birth: dd |_|_| mm |_|_| yyyy |_|_|_|_|
2. Birth place:

City/town _______________________________________ |_|_|_|_|_|_|

Province _____________________________________________ |_|_|_|_|_|_|

ZIP code |_|_|_|_|_|_|

Country of birth _______________________________________ |_|_|_|_|_|_|

1. Marital status:

1. married | cohabitant

2. widow

3. separate | divorced

4. single | never married

1. Level of education:

1. none

2. elementary school

3. middle school

4. High School

5. University

1. Current occupational status:

1. employed on maternity leave

2. currently employed

3. housewife

4. unemployed

5. Other conditions (specify) ___________________________ |_|_|_|_|

1. Please indicate

your job title ___________________________________________________ |_|_|_|_|

your occupational sector _________________________________________ |_|_|_|_|

| 7. Your occupation is: |
| --- |
| 1. armed forces occupations |
| 1. manager |
| 1. professional |
| 1. technicians and associate professionals |
| 1. clerical support workers |
| 1. service and sales workers |
| 1. craft and related trades workers |
| 1. plant and machine operators, and assemblers |
| 1. elementary occupations |

**Health behaviours and conditions**

1. Please indicate if you had the following comorbidities before or during this pregnancy:

|  | No, never | Yes, only during pregnancy | Yes, just before pregnancy | Yes, both before and during pregnancy |
| --- | --- | --- | --- | --- |
| diabetes | 1 | 2 | 3 | 4 |
| asthma | 1 | 2 | 3 | 4 |
| allergy | 1 | 2 | 3 | 4 |
| epilepsy | 1 | 2 | 3 | 4 |
| hypertension | 1 | 2 | 3 | 4 |
| vomit | 1 | 2 | 3 | 4 |
| hypothyroidism | 1 | 2 | 3 | 4 |
| hyperthyroidism | 1 | 2 | 3 | 4 |
| lupus | 1 | 2 | 3 | 4 |
| rheumatic diseases | 1 | 2 | 3 | 4 |
| urinary infections | 1 | 2 | 3 | 4 |
| infections | 1 | 2 | 3 | 4 |
| fever | 1 | 2 | 3 | 4 |
| seizures | 1 | 2 | 3 | 4 |
| anemia | 1 | 2 | 3 | 4 |
| cardiovascular diseases | 1 | 2 | 3 | 4 |
| neurological diseases | 1 | 2 | 3 | 4 |

1. Have you ever taken medications – on a regular basis - during this pregnancy?

1. Yes

2. No

1. Which medications have you used during pregnancy? Please list the commercial name of each medication, active substance, if known, and its indication

|  | commercial name | active substance | indication |
| --- | --- | --- | --- |
| 1 |  |  |  |
| 2 |  |  |  |
| 3 |  |  |  |
| 4 |  |  |  |
| 5 |  |  |  |
| 6 |  |  |  |

1. How many gynecological - obstetric visits did you undergo during this pregnancy ? |_|_|
2. How many ultrasounds examinations did you undergo during this pregnancy? |_|_|
3. Are you a smoker?

1. No, I have never smoked

2. Yes, I am a current smoker

3. I am an ex-smoker: I quit smoking before this pregnancy

4. I am an ex-smoker: I quit smoking during this pregnancy at the | _ | month

5. I am an ex-smoker: I quit smoking after this pregnancy

1. If you are a current or ex-smoker, at what age did you start smoking? |_|_| years
2. If you are an ex-smoker, at what age did you stop smoking? |_|_| years

**Data of the baby**

1. Date of birth : dd |_|_| mm |_|_| yyyy |_|_|_|_|
2. Gestational age at birth (weeks) : |_|_|
3. Sex

1. Male

2. Female

1. Birth weight gr |_|_|_|_|
2. Birth length cm |_|_|

End of questionnaire completion dd |_|_| mm |_|_| yyyy |_|_|_|_|
