# Supplementary material for: Medication use during pregnancy, gestational age and date of delivery: agreement between maternal self-reports and health database information in a cohort
Source: BMC Pregnancy Childbirth. 2015 Nov 25;15:310. doi: 10.1186/s12884-015-0745-3 (PMC4660837; doi:10.1186/s12884-015-0745-3)
Supplement: Additional file 2: — Supplemental tables. Table S1. Agreement between questionnaire and prescription redemption database for selected therapeutic classes by time of questionnaire completion. Table S2. Number of women with information on hypertension during pregnancy and agreement between questionnaire and birth certificate database. Table S3. Number of women with information on hypertension during pregnancy in questionnaire and in birth certificate database and use of antihypertensive medications according to questionnaire and prescription database. Positive Predictive Value and Negative Predictive Value of prescriptions for antihypertensive medications recorded in questionnaire and in birth certificate database. (DOCX 23 kb) [file 12884_2015_745_MOESM2_ESM.docx]

| Table S1. Agreement between questionnaire and prescription redemption database for selected therapeutic classes by time of questionnaire completion. | | | | | | | | | |
| --- | --- | --- | --- | --- | --- | --- | --- | --- | --- |
|  | Time of questionnaire completion | | | | | | | | |
|  | Pre-delivery (N=402) | | | |  | Post-delivery (N=357) | | | |
| Therapeutic class | Kappa | 95%CI | Strength of agreement^1^ | PABAK |  | Kappa | 95%CI | Strength of agreement^1^ | PABAK |
| folic acid | 0.10 | 0.00; 0.20 | slight | 0.23 |  | 0.12 | 0.02; 0.23 | slight | 0.20 |
| iron | 0.46 | 0.36; 0.56 | moderate | 0.59 |  | 0.52 | 0.42; 0.61 | moderate | 0.60 |
| non-opioid analgesics | -0.005 | -0.01; 0.00 | poor | 0.90 |  | -0.005 | -0.02; 0.00 | poor | 0.83 |
| thyroid hormones | 0.89 | 0.77; 1.00 | almost perfect | 0.98 |  | 0.88 | 0.77; 0.987 | almost perfect | 0.97 |
| antacids | 0.20 | -0.02; 0.41 | slight | 0.89 |  | 0.16 | -0.06; 0.38 | slight | 0.90 |
| antithrombotic agents | 0.73 | 0.54; 0.92 | substantial | 0.96 |  | 0.68 | 0.48; 0.87 | substantial | 0.95 |
| antibiotics, systemic^1^ | 0.05 | -0.02; 0.12 | slight | 0.59 |  | 0.198 | 0.07; 0.31 | slight | 0.68 |
| progestogens | 0.26 | 0.12; 0.39 | fair | 0.78 |  | 0.318 | 0.15; 0.48 | fair | 0.81 |
| antihypertensive medications | 1.00 | 1.00; 1.00 | almost perfect | 1.01 |  | 0.838 | 0.60; 1.00 | almost perfect | 0.99 |
| medications for obstructive airway disease | 0.24 | -0.04; 0.52 | fair | 0.95 |  | 0.29 | 0.03; 0.55 | fair | 0.93 |
| ^1^ According to Landis and Koch [22]. | | | | | | | | | |

| Table S2. Number of women with information on hypertension during pregnancy and agreement between questionnaire and birth certificate database. | | | | | | | |
| --- | --- | --- | --- | --- | --- | --- | --- |
|  |  | birth certificate database | | Kappa | 95%CI | Strength of Agreement^2^ | PABAK |
| questionnaire^1^ |  | yes | no |  |  |  |  |
| yes |  | 10 | 23 | 0.40 | 0.22; 0.58 | fair | 0.92 |
| no |  | 5 | 664 |  |  |  |  |
| Total |  | 15 | 687 |  |  |  |  |

^1^ A total of 63 women had missing information.

^2^ According to Landis and Koch [22].

| Table S3. Number of women with information on hypertension during pregnancy in questionnaire and in birth certificate database and use of antihypertensive medications according to questionnaire and prescription database. Positive Predictive Value and Negative Predictive Value of prescriptions for antihypertensive medications recorded in questionnaire and in birth certificate database. | | | | | | | | | | | |
| --- | --- | --- | --- | --- | --- | --- | --- | --- | --- | --- | --- |
|  |  | Antihypertensive medications | | | | |  | Predictive values of prescriptions  for antihypertensive medications | | | |
|  |  | questionnaire | |  | prescription database | |  |  |  |  |  |
| hypertension |  | yes | no |  | yes | no |  | Positive Predictive Value | 95%CI | Negative Predictive Value | 95%CI |
| questionnaire^1^ |  |  |  |  |  |  |  |  |  |  |  |
| yes |  | 6 | 27 |  | 8 | 25 |  | 100 | 100; 100 | 96.4 | 95.0; 97.8 |
| no |  | 0 | 669 |  | 0 | 669 |  |  |  |  |  |
|  |  |  |  |  |  |  |  |  |  |  |  |
| birth certificate database |  |  |  |  |  |  |  |  |  |  |  |
| yes |  | 6 | 10 |  | 8 | 8 |  | 100 | 100; 100 | 98.9 | 98.2; 99.6 |
| no |  | 0 | 751 |  | 0 | 751 |  |  |  |  |  |

^1^ A total of 63 women had missing information.
